# Impact of obesity on global and regional systolic function in children: a CMR study

**DOI:** 10.1186/1532-429X-13-S1-P310

**Published:** 2011-02-02

**Authors:** Magalie Viallon, Julie Wacker, Patrick Clarysse, Maurice Beghetti, Nathalie Farpour-Lambert, Pierre Croisille

**Affiliations:** 1 Radiology department, Hopital Universitaire de Genève, Geneva, Switzerland; 2 Pediatric Cardiology Unit, Child and adolescents department, Hopital Universitaire de Genève, Geneva, Switzerland; 3 CREATIS-LRMN ; CNRS UMR5220 ; INSERM U630 ; INSA-Lyon ; Cardiology Hospital L. Pradel, Lyon University, Lyon, France

## Introduction

Early detection of subclinical manifestations of obesity is important to initiate early effective treatment aiming at reversing the process. Recent studies using echocardiography tissue doppler or strain rate imaging have suggested the potential of motion tracking methods to characterize subtle systolic changes that may be associated with obesity in children (1,2).

## Purpose

The aim of the present study was to investigate LV global and intra-myocardial regional systolic mechanics using CMR tagging in obese and non-obese adolescents in order to better characterize the subclinical early changes associated with obesity in children.

## Methods

Standard CMR global and regional LV function study and cine-tagging with complementary myocardial tagging(CSPAMM) were performed in 25 obese children and 25 lean controls. Inclusion criteria were: no previous diagnosis of hypertension and treatment with antihypertensive/anti-diabetic drugs or drugs known to affect glucose and lipid metabolism; no history of familial hypertension/dyslipidemia; absence of diabetes or other chronic disease. The study protocol has been approved by the Mother&Child Ethics Committee. Magnitude CSPAMM images were processed using *InTag* post-processing toolbox (Creatis, Lyon, France) implemented in OsiriX software (Geneva, Switzerland) to perform quantitative myocardial strain analysis. Motion estimation is based on the *Sine Wave Modeling* approach(3). Regional and global peak circumferential(E_cc_), longitudinal(E_ll_) strains as well as peak rotation and torsion were calculated.

## Results

Gender distribution was not different between the two groups but obese children were older(14.1±1vs 12.9±1.7yrs,p<0.01). BMI(30.4±5.4vs18.9±1.9),Z-score(2.58vs0.02,p<0.0001), systolic (112±13vs101±10 mmHg,p<0.01) and diastolic blood pressure (55±6vs52±7mmHg, p<0.05) were greater whereas heart rate(71±9vs77±9bpm,p<0.05) was lower in the obese group. Measurements of LV mass (97.24±21.2vs80.5±20.2g,p<0.0001) and EDV (117.6±32.6vs101.3±23.7 ml,p<0.01) were significantly greater in the obese children. Although global systolic function was not different among the two groups, obese children exhibited a significantly increased stroke volume (60.44±6.74vs 58.06±5.7ml,p<0.01). In obese children, E_cc_ displayed only a trend to lower values in basal level (-0.15±0.2 vs -0.16±0.2%,p=NS) similarly to what was observed for E_ll_ (-0.14±0.2 vs 0.15±.01,p=NS). Torsion was also not significantly increased in obese children (19.5±3.8° vs 18.0±4.3°,p=NS).

## Conclusion

If our data confirm that obese children have early morphological changes in LV mass, EDV and SV, regional circumferential and longitudinal strains are not yet altered from normal. The trend to increased torsion and increased wall thickness may be an adaptation to increased LV ED volume and may lead to early impairment of LV diastolic function.

## References

[B1] Di Salvo G EurHeartJ 2006 10.1093/eurheartj/ehl163 16905554

[B2] Lorch SM JCardiometabSyndr 2007

[B3] ArtsT.IEEETransMedImaging 2010

